# Frequency reallocation based on cochlear place frequencies in cochlear implants: a pilot study

**DOI:** 10.1007/s00405-021-07245-y

**Published:** 2022-01-15

**Authors:** Flavia Di Maro, Marco Carner, Andrea Sacchetto, Davide Soloperto, Daniele Marchioni

**Affiliations:** 1 Otolaryngology-Head and Neck Surgery Department, University Hospital of Verona, Piazzale Aristide Stefani, 1, 37126 Verona, Italy; 2 Otolaryngology-Head and Neck Surgery Department, University of Verona, Via S. Francesco, 22, 37129 Verona, Italy

**Keywords:** Frequency reallocation, Frequency-to-place mismatch, Cochlear implant, Anatomy-based fitting

## Abstract

**Purpose:**

The aim of this study is to evaluate speech perception outcomes after a frequency reallocation performed through the creation of an anatomically based map obtained with Otoplan^®^, a tablet-based software that allows the cochlear duct length to be calculated starting from CT images.

**Methods:**

Ten postlingually deafened patients who underwent cochlear implantation with MED-EL company devices from 2015 to 2019 in the Tertiary referral center University Hospital of Verona have been included in a retrospective study. The postoperative CT scans were evaluated with Otoplan^®^; the position of the intracochlear electrodes was obtained, an anatomical mapping was carried out and then it was submitted to the patients.

All patients underwent pure tonal and speech audiometry before and after the reallocation and the audiological results were processed considering the Speech Recognition Threshold (SRT), the Speech Awareness Threshold (SAT) and the Pure Tone Average (PTA). The differences in the PTA, SAT and SRT values before and after the reallocation were determined. The results were statistically processed using the software Stata with a significance value of *α* < 0.05.

**Results:**

The mean values of SRT (61.25 dB versus 51.25 dB) and SAT (49 dB versus 41 dB) were significantly lower (*p*: 0.02 and *p*: 0.04, respectively) after the reallocation. No significant difference was found between PTA values (41.5 dB versus 39.25 dB; *p*: 0.18).

**Conclusions:**

Our preliminary results demonstrate better speech discrimination and rapid adaptation in implanted postlingually deaf patients after anatomic mapping and subsequent frequency reallocation.

## Introduction

At the time of activation of a cochlear implant (CI), one of the parameters set is the spectral distribution in which the frequencies of the incoming sounds, once processed by the processor, are delivered by the intracochlear electrodes. In clinical practice, for each implant model, a default frequency distribution is usually assigned along the array. Given the inter-individual anatomic variability of the Cochlear Duct Length (CDL), this generalized approach could generate a mismatch between the frequency assigned to the electrode and the characteristic frequency of the neurons that it stimulates. This mismatch in patients with post-lingual deafness creates distortions due to the difference between the frequency perceived with the CI and the characteristics of the sound that they would expect to hear based on their auditory memory [1].

The frequency distribution at the level of the basilar membrane follows a logarithmic law, known as the Greenwood function. Knowing the CDL, this function allows the position of the hair cells in the Organ of Corti (OC) to be related to the frequencies that stimulate the corresponding auditory neurons [2]. Historically, one of the major limitations in the application of the Greenwood function for the determination of the frequency distribution at the level of the basilar membrane was the impossibility of measuring the exact CDL starting from the preoperative radiology.

Otoplan^®^ is a tablet-based software designed by CAScination AG (Bern, Switzerland) in collaboration with the MED-EL Corporation (Innsbruck, Austria) that allows the CDL to be calculated accurately starting from CT images [3]. Through the reconstruction generated by the software, it is possible to establish the electrode that guarantees the greatest cochlear coverage for the patient.

After surgery, having a high-resolution CT image available and identifying the position of each electrode inserted, it is possible to locate their insertion depths with extreme precision [4]. This makes it possible to apply the Greenwood function in the correlation between the frequency assigned to a determined electrode and the characteristic frequency of the neural element stimulated through it. From this analysis, an anatomically based mappingis is obtained, that proposes a signal to the neural elements as close as possible to what they perceived before the hearing loss, in an attempt to reduce the adaptation times to improve the speech discrimination performances of patients with post-verbal deafness.

The purpose of this study is to demonstrate that an anatomically based frequency reallocation can provide immediate benefit to the patient, reducing the time required for adaptation and allowing final performances to be achieved which are better than the standard approach with a default frequency distribution.

## Materials and methods

A consultation of the surgical database held by the ENT department in the University Hospital of Verona was carried out, selecting all patients who underwent cochlear implantation between January 2015 and December 2019. Inclusion criteria were: cochlear implants manufactured by MED-EL Corporation and postlingually deaf patients aged > 14 years, as this age group presents the possibility of comparing the subjective map with the anatomy-based one and the presence of auditory memory. The minimum accepted CT definition was a slice thickness of 1 mm. Exclusion criteria were: diagnosis of cochlear malformations or cochlear ossification; cochlear implants performed after infrapromontorial approaches for acoustic neuroma; patients with prelingual deafness or under the age of 13; patients lost to follow-up or whose radiology was not available or with inadequate definition at the time of the study. Another crucial condition, necessary for the anatomy-based fitting, was the presence of two electrodes corresponding to frequencies below 950 Hz, two electrodes between 950 and 3000 Hz and two electrodes for frequencies above 3000 Hz.

All patients underwent pure tone and speech audiometry before the frequency reallocation and the audiological results were processed considering the Speech Recognition Threshold (SRT), the Speech Awareness Threshold (SAT) and the Pure Tone Average (PTA), the average of pure tone hearing thresholds at 500, 1000, 2000 and 4000 Hz.

The default frequency values assigned to each electrode during normal subjective mapping were recorded for each patient. The postoperative CT scans were evaluated with Otoplan^®^, determining the array insertion depth and the subsequent cochlear place of stimulation.

**Fig. 1:**
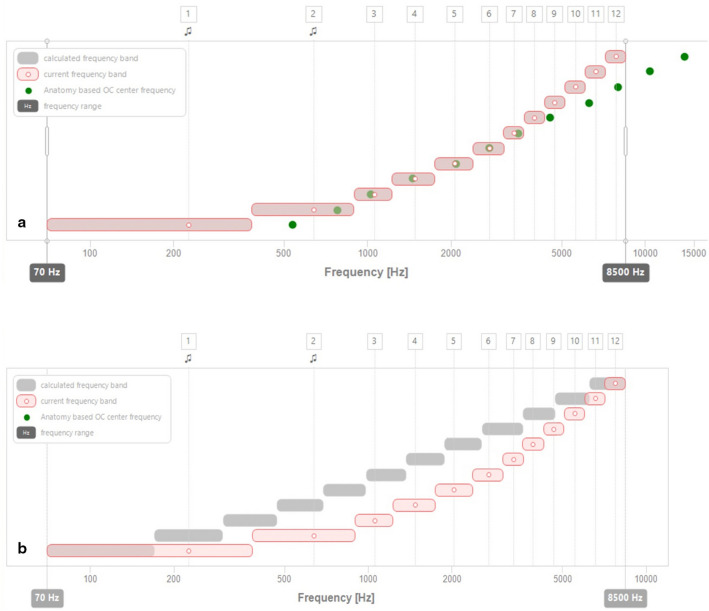
**a** Anatomically based mapping of a patient (P2) included in the study. Dotted line: characteristic frequencies of the neural elements stimulated by each electrode. Rectangles: frequency bands of the new map based on anatomic information. **b** Frequency reallocation in the same patient: comparison between the default frequencies distribution assigned during subjective mapping (darker rectangles) and between the frequency distribution obtained on an anatomic basis (clearer rectangles)

With this anatomic information, MAESTRO 9.0 software (MED-EL) automatically elaborates a new frequency map on an anatomical basis (Fig. 1). The Otoplan^®^ software has an automated frequency allocation formula that, at this stage, does not include any variability of manual reallocation. The MAESTRO 9.0 software allows manual shift but, in this preliminary study, we have not used this feature: we have purely applied the Otoplan^®^ formula, as set by the manufacturer.

The reallocation formula focuses on minimizing the mismatch on a specific frequency band: 950–3000 Hz. The MED-EL company has made this initial choice to optimize the match in the most important frequencies for speech perception. Moreover, below 950 Hz, MED-EL implants use Fine Structure coding and the assumption is that, for those frequencies, rate coding should be more prominent than place coding. Therefore, the software optimizes the frequency allocation between 950 and 3000 Hz and spreads the mismatch for lower and higher frequencies. Other principles on which frequency reallocation is based are:

- The overall frequency band is maintained as before (usually 70–8500 Hz) and not reduced to minimize mismatch.
- No electrodes are deactivated (none of them was extracochlear) to reduce mismatch in case they are outside the full frequency band mentioned above (e.g., a basal electrode in the cochlea but placed at 12.000 Hz).

The new maps thus obtained were administered to the patients who expressed their subjective sensations after the frequency reallocation in terms of improvement or worsening of the sound sensation and how the perceived pitch had changed after a usage time of 30 min. The patients were tested with pure tone and speech audiometry after the reallocation. The statistical significance of the difference in the PTA, SAT and SRT thresholds obtained before and after the frequency reallocation was considered. The correlation between the array depth of insertion and those audiological thresholds was evaluated.

**Fig. 2:**
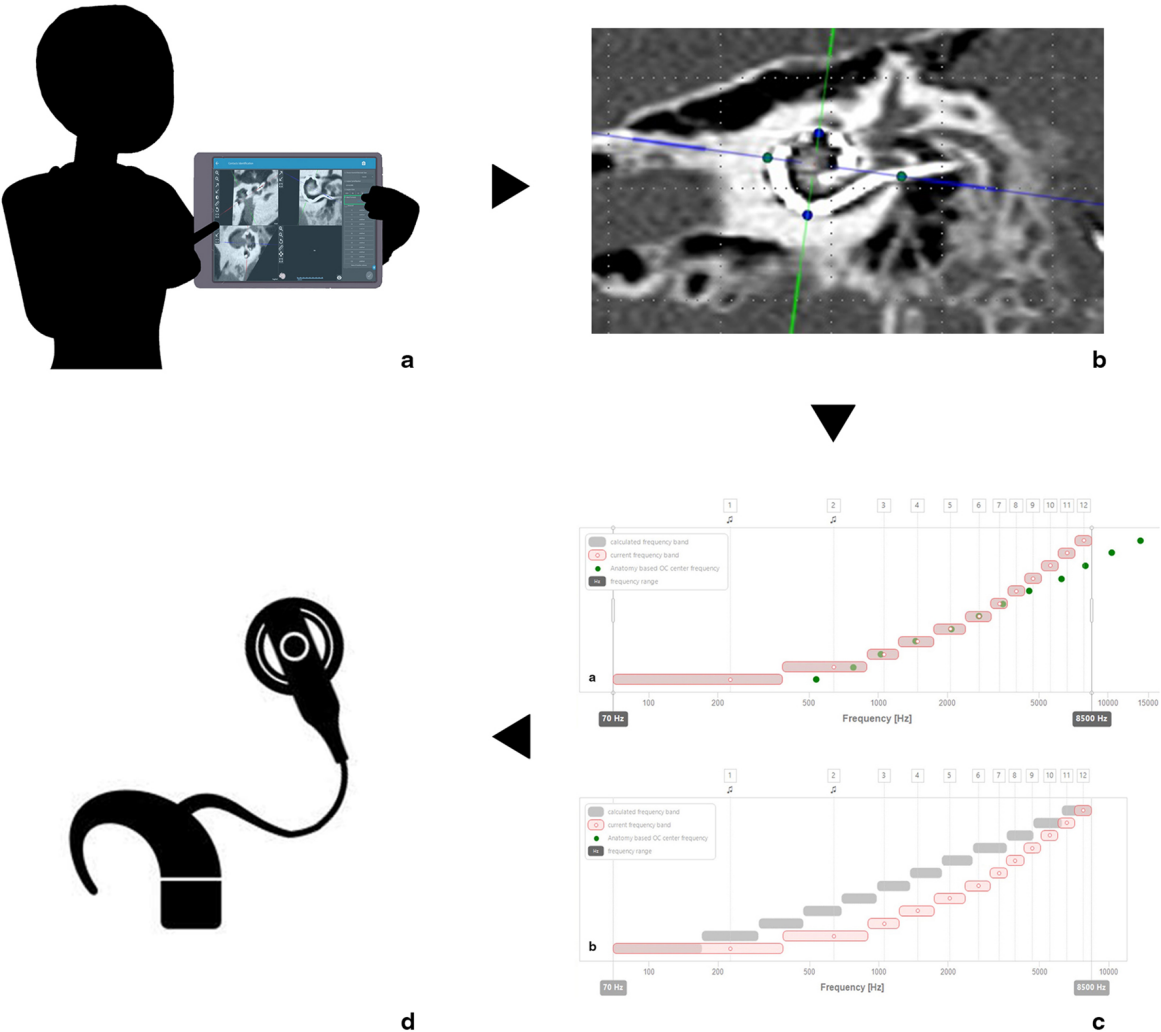
A schematic illustration on the use of Otoplan^®^. **a** The user identifies some cochlear parameters on a three-dimensional view of the cochlea elaborated by the program starting from the CT scan (the modiolus, the round window, the lateral, upper and lower limits of the cochlea) to calculate the length of the DC according to the method of elliptic approximation. **b** Having a high-resolution CT scan available and identifying (manually or using automatic identification) the position of each electrode inserted, it is possible to obtain the insertion depth of each of them with extreme precision, making it possible to apply the Greenwood function in the correlation between the frequency assigned to a given electrode and the characteristic frequency of the neural element stimulated by it. **c** From this analysis, the MAESTRO 9.0 software obtains a mapping on an anatomical basis (see Fig. 1) that is proposed to the patient (**d**) in place of his previous frequency map, assigned by default

Results were obtained using Student's *t *test. Statistical analysis was performed using Stata software (Stata Corporation, College Station, TX, USA). The use and principles of Otoplan^®^ have been schematized in Fig. 2.

The study was approved by the local IRB and informed consent was obtained from all patients.

## Results

Ten patients (3 female, 7 male) were included in the study and all their individual data are reported in Table 1. Their mean age was 55 years (14.3–78.7 years). The time elapsed from activation of the CIs at the time of the study was on average 22.4 months (9.8–61.3 months). The time elapsed between postoperative CT and frequency reallocation varies between 31 and 60 days (on average 46 days).

**Table 1:** Individual data of the patients included

	Age (years)	Sex	PTApre (dBHL)	PTApost (dBHL)	SRTpre (dBHL)	SRTpost (dBHL)	SATpre (dBHL)	SATpost (dBHL)	Time between the reallocation and final audiometry (days)	CDL (mm)	Array	Time from implantation (months)	Central frequency of the first electrode (Hz)	Array insertion depth
P1	62	M	38,75	40	–	–	65	65	33	40.4	FLEX28	31.2	299	564.5°
P2	14	M	42,5	32,5	65	50	45	35	39	39.7	FLEX28	61.3	534	464.5°
P3	78	M	43,75	41,25	55	55	35	45	42	44.6	FLEX28	11.9	445	508.9°
P4	57	F	58,75	40	85	50	75	35	76	42.5	FLEX28	9.8	493	480.6°
P5	56	F	38,75	40	55	55	40	35	33	40.3	FLEX28	19.7	315	555.9°
P6	57	M	38,75	37,5	55	50	40	35	72	44.6	FLEX28	17.2	332	548.5°
P7	45	M	42,5	45	50	45	40	35	25	46.8	FLEX28	22.5	538	463.2°
P8	74	F	37,5	42,5	60	50	35	35	33	37.5	FLEX28	16.3	304	560.5°
P9	55	M	32,5	28,75	65	55	55	45	30	38.8	FLEX28	31.2	282	568°
P10	48	M	41,25	45	–	–	60	45	33	38.2	FLEXSOFT	2.5	310	564.8°

*PTA* Pure Tone Average, *SRT* Speech Recognition Threshold, *SAT* Speech Awareness Threshold, − pre preoperative, − *post* postoperative, *CDL* Cochlear Duct Length

Pure tone and speech audiometry was performed on average 41.6 days after the reallocation of frequencies (25–76 days).

Nine out of the ten patients had a Flex 28 array (90%), while one patient (10%) had a FLEXSOFT array. All of the patients presented complete insertion.

The central frequency of the first intracochlear electrode was on average 385.2 Hz (282–538 Hz) and the angular depth of insertion was on average 527.9° (463.2–568°).

The software-estimated CDL averaged 41.3 mm (37.5–46.8 mm).

The audiological results are shown in Table 2. The differences between the values of PTA, SAT and SRT before and after the reallocation were investigated and the results showed a statistically significant difference between the SRT and the SAT values before and after reallocation (*p*: 0.02 and *p*: 0.04, respectively). In fact, the SRT and SAT values before the reallocation appeared to be higher than that after the anatomic mapping; however, there is no statistically significant difference between the PTA thresholds. As shown in Table 2, two of the patients did not reach the SRT, even before the frequency reallocation, and that threshold was then not available. Increasing electrode insertion depth correlates with an improvement trend of the audiometric thresholds but with no statistical significance (PTA: *R*^2^ = 0.09, *p* = 0.40; SRT: *R*^2^ = 0.49, *p* = 0.05; SAT: *R*^2^ = 0.33, *p* = 0.08).

**Table 2:** Comparison *t *test between the means of PTA, SRT and SAT before (pre) and after (post) the frequency reallocation

	Mean (dB)	Student’s *t *test	Pr
PTA pre	41.5	t: 0.98 (9 degrees of freedom)	Pr (T > t): 0.17
PTA post	39.25		
SRT pre	61.25	t: 2.49 (7 degrees of freedom)	Pr (T > t): 0.02
SRT post	51.25		
SAT pre	49	t: 1.92 (9 degrees of freedom)	Pr (T > t): 0.04
SAT post	41		

*Pr* *p *value, *T* Standardized *T* score, *t* Student *t *statistic, *PTA* Pure Tone Average, *SAT* Speech Awareness Threshold, *SRT* Speech Recognition Threshold (SRT)

After the reallocation, half of the patients (5/10) perceived an immediate subjective improvement in the quality of auditory perception; 3/10 patients stated that the sound was different, but they could not tell if there had been an improvement or a worsening of its quality; 2/10 perceived a deterioration in sound quality after reallocation. All of the patients (10/10) noted that their perception of the pitch of sounds was low-pitched after the anatomic mapping.

## Discussion

Speech discrimination in patients undergoing cochlear implantation is influenced by numerous non-modifiable factors, such as the age at the time of surgery, duration of hearing deprivation and the health of the cochlea [5].

The association between insertion depth of the arrays and postoperative speech discrimination has been demonstrated in numerous studies [6–8] which have sparked interest in the development of new and more precise methodologies for measuring the insertion depth of the electrodes and the CDL. Using this software, it is currently possible to optimize the correspondence between the frequency assigned to an electrode and the characteristic frequency of the neural element stimulated using two steps: (i) in the preoperative phase, through the study of a patient’s CT scan, it is possible to obtain 3D multiplanar cochlear reconstructions, allowing estimation of the CDL and so to choose the array capable to reach the greatest cochlear coverage for that patient; (ii) in the postoperative period, through the study of postoperative CT scans, the software allows the position of each electrode to be detected with great accuracy at the level of the cochlea, estimating the characteristic frequency of the neurons stimulated it using the Greenwood function [4]. From this data, the mapping software generates a map on an anatomical basis by matching, with extreme precision, the central frequencies typical of speech (between 950 and 3000 Hz) and tolerating mismatch at low and high frequencies (Fig. 1).

In the present study, Otoplan^®^ has only been used for postoperative remapping of previous implanted patients to demonstrate that a better correspondence between the anatomical frequency distribution and the frequency bands of the electrodes results in a better speech discrimination.

The reliability of Otoplan^®^ in determining the CDL and the depth of insertion of the array has been demonstrated in earlier studies [4] but the clinical implications of what has been demonstrated have not yet been thoroughly investigated.

Canfarotta et al. [9] found that as the age of patients and the frequency-to-place mismatch present at 1500 Hz increased, the patients’ speech discrimination deteriorated. Given the auditory processing deficits associated with advanced age, they hypothesized that this class of patients would benefit most from a reduction in the mismatch.

In accordance with these considerations, in our study, those with the poorest results were the older patients, probably as a consequence of their reduced neuronal plasticity. Arguably, a preoperative study of the cochlear length and consequent customization of the choice of array, minimizing the need for adaptation, could lead to a significant benefit in these patients, as well as in pediatric patients, where the default mapping is based on imprecise parameters.

This pilot study explores the clinical implications of applying anatomic mapping with Otoplan^®^ in patients with cochlear implants. In our patients, after the reallocation, the SRT and SAT were significantly lower, proving a significant improvement in speech discrimination (*p*: 0.02 and *p*: 0.04, respectively).

The audiological data related to the frequency reallocation were collected an average of 41.6 days (25–76 days) after the reallocation. Therefore, there was minimal time for adaptation and no adjustment as follows normal subjective mapping (adaptation and modifications that instead led to the production of the map previously used by the patients), but the results were, on average, still better than those before the reallocation.

Finally, the patients who had undergone frequency reallocation reported the perception of sounds as low-pitched; this is explainable by considering that the acoustic neurons in these patients were stimulated at frequencies much higher than their characteristic frequency in relation to the shift of the frequency range towards the higher pitch of the cochlear basal turn, defined in previous studies as basalward shift [1, 10] related to the failure of the array electrodes to reach greater insertion depths.

When analyzing the patients’ map (for example in Fig. 1), we studied the position of the cochlear electrodes (dots). Their position on the basilar membrane implies that the neurons stimulated by these electrodes have frequencies which are much higher than the default frequencies assigned to the electrodes to cover the entire frequency range available (darker rectangles). Taking for example the patient in the figure (P2 in Table 1), before the reallocation, a sound of 1000 Hz was delivered by the sixth electrode of the array (with a central frequency of 1175 Hz); according to the anatomic reconstruction, this sound was perceived by auditory neurons with a characteristic frequency of about 2500 Hz. After the reallocation, a frequency of 1000 Hz was delivered by the third electrode (with a central frequency of 1056 Hz) which stimulates a neuron with approximately the same characteristic frequency, thus determining the sensation of perceiving a low-pitched sound.

In addition, in MED-EL cochlear implants, the electrodes with assigned frequency bands below the value of 1000 Hz stimulate the nerve using the same frequency as the input sound, to recreate the phase locking that occurs under physiological conditions. Following the frequency reallocation, as seen in Fig. 1, the electrodes responsible for delivering the low-pitched frequencies cover a wider frequency band than the previous map, and this may give the sensation of low-pitched sounds.

To our knowledge, this is the first study describing audiological findings after an anatomically based frequency reallocation in patients previously undergoing cochlear implantation. However, this is a pilot study, and further investigations involving a greater number of patients and a longer follow-up will more solidly demonstrate the significance of the results obtained. The present work is a clinical study based on previous conclusions drawn from other valuable studies using audiological tests in quiet [7, 9]. Nevertheless, given the good results obtained with these simple tests, familiar to the patients and comparable with the literature, further evaluation of the speech recognition in noise after the reallocation it would be appropriate in future.

In conclusion, the preliminary results of this study show that performing a frequency reallocation based on the cochlear position of the electrodes in implanted patients results in better speech discrimination compared to standard mapping in the same patients. The achievement of better audiological results compared to those obtained after months or years of adaptation and numerous fitting sessions, makes this mapping method potentially useful for all implanted patients. Theoretically, anatomic mapping could improve results, especially when it is difficult to obtain subjective feedback (for example, in pediatric patients) or in those cases in whom reduced neuronal plasticity makes the adaptation process long and laborious (for example, in elderly patients, if the reallocation is preceded by a preoperative study and consequent choice of the custom array).
